# Caveolin-1 Plays a Crucial Role in Inhibiting Neuronal Differentiation of Neural Stem/Progenitor Cells via VEGF Signaling-Dependent Pathway

**DOI:** 10.1371/journal.pone.0022901

**Published:** 2011-08-03

**Authors:** Yue Li, Jianmin Luo, Wui-Man Lau, Guoqing Zheng, Shuping Fu, Ting-Ting Wang, He-Ping Zeng, Kwok-Fai So, Sookja Kim Chung, Yao Tong, Kejian Liu, Jiangang Shen

**Affiliations:** 1 School of Chinese Medicine, The University of Hong Kong, Hong Kong SAR, China; 2 Research Centre of Heart, Brain, Hormone and Healthy Aging, The University of Hong Kong, Hong Kong SAR, China; 3 Center of Neurology and Rehabilitation, The Second Affiliated Hospital of Wenzhou Medical College, Wenzhou, China; 4 Institute of Functional Molecule, School of Chemistry, South China University of Technology, Guangzhou, China; 5 State Key Laboratory of Brain and Cognitive Sciences, Department of Anatomy, The University of Hong Kong, Hong Kong SAR, China; 6 Center of Biomedical Research Excellence, College of Pharmacy, University of New Mexico, Albuquerque, New Mexico, United States of America; Center for Regenerative Therapies Dresden, Germany

## Abstract

In the present study, we aim to elucidate the roles of caveolin-1(Cav-1), a 22 kDa protein in plasma membrane invaginations, in modulating neuronal differentiation of neural progenitor cells (NPCs). In the hippocampal dentate gyrus, we found that Cav-1 knockout mice revealed remarkably higher levels of vascular endothelial growth factor (VEGF) and the more abundant formation of newborn neurons than wild type mice. We then studied the potential mechanisms of Cav-1 in modulating VEGF signaling and neuronal differentiation in isolated cultured NPCs under normoxic and hypoxic conditions. Hypoxic embryonic rat NPCs were exposed to 1% O_2_ for 24 h and then switched to 21% O_2_ for 1, 3, 7 and 14 days whereas normoxic NPCs were continuously cultured with 21% O_2_. Compared with normoxic NPCs, hypoxic NPCs had down-regulated expression of Cav-1 and up-regulated VEGF expression and p44/42MAPK phosphorylation, and enhanced neuronal differentiation. We further studied the roles of Cav-1 in inhibiting neuronal differentiation by using Cav-1 scaffolding domain peptide and Cav-1-specific small interfering RNA. In both normoxic and hypoxic NPCs, Cav-1 peptide markedly down-regulated the expressions of VEGF and flk1, decreased the phosphorylations of p44/42MAPK, Akt and Stat3, and inhibited neuronal differentiation, whereas the knockdown of Cav-1 promoted the expression of VEGF, phosphorylations of p44/42MAPK, Akt and Stat3, and stimulated neuronal differentiation. Moreover, the enhanced phosphorylations of p44/42MAPK, Akt and Stat3, and neuronal differentiation were abolished by co-treatment of VEGF inhibitor V1. These results provide strong evidence to prove that Cav-1 can inhibit neuronal differentiation via down-regulations of VEGF, p44/42MAPK, Akt and Stat3 signaling pathways, and that VEGF signaling is a crucial target of Cav-1. The hypoxia-induced down-regulation of Cav-1 contributes to enhanced neuronal differentiation in NPCs.

## Introduction

Neural progenitor or stem cells (NPCs) can potentially generate new functional neurons for brain repair in the adult central nervous system (CNS) [1], [2]. Neurogenesis can be divided into several stages including proliferation, maturation, differentiation and integration, etc which mainly occur in two areas of adult brains, that is, the subventricular zone (SVZ) lining the lateral ventricle and the subgranular zone (SGZ) in the dentate gyrus of hippocampus [3], [4]. The division and differentiation of these endogenous NSCs can be regulated by physiological stimuli and pathological conditions [5]. Enhanced neurogenesis has been reported in hypoxic NPCs *in vitro* [6], [7] and in ischemic brains of neonatal mice [8], adult rats [9] and aged humans *in vivo* [10]. For examples, exposure to 1–5% oxygen concentration improved NPCs proliferation, survival and dopaminergic neuron development [11], [12]. Mild hypoxia (2.5 to 5% oxygen) remarkably promoted proliferation and differentiation of human neural stem cells into neuro-oligodendroglial progenitors [13]. Cerebral ischemia increased neurogenesis and promoted the normal development of newly generated neurons in the adult dentate gyrus (DG) [14], [15]. However, the mechanisms of hypoxia/ischemia-induced neurogenesis are largely unknown.

Vascular endothelial growth factor (VEGF) plays a crucial role in neurogenesis [16], [17]. VEGF exerts its action via phosphotyrosine kinase receptors including VEGFR1/fms-like tyrosine kinase (flt) and fetal liver kinase1 (flk1). VEGF can activate divergent intracellular signaling components and regulate retinal progenitor cell proliferation and neuronal differentiation. VEGF can also mediate the cross-talk between neural stem cells and endothelial cells [18], [19]. Transient oxygen and glucose deprivation (OGD) treatment enhanced VEGF signaling via regulation of hypoxia-inducible factor 1α (HIF-1α) transcription factor [20]. Hypoxia induced the up-regulation of VEGF and flk1 and stimulated the proliferation and differentiation of adult NPCs *in vitro* and promoted neurogenesis *in vivo* [21], [22]. VEGF was found to enhance neurogenesis, neuromigration, angiogenesis, and to improve the recovery of neurological deficits and learning ability in the animal models of focal cerebral ischemic injury and spinal cord injury [23], [24], [25], [26]. Thus, VEGF signal pathway is critical in brain repair after ischemic brain injury.

VEGF-induced extracellular signal-regulated kinase (ERK) or p44/42 mitogen-activated protein kinase (p44/42MAPK) signaling pathways participate in neurogenesis [27]. Hypoxia-reoxygenation can up regulate VEGF expression and activate p44/42MAPK signaling pathways in NPCs [20], [28]. Flk1, a VEGF receptor, mediates the activation of p44/42MAPK and subsequently promotes neurite extension in peripheral or central neurons [29], [30]. The activation of p44/42MAPK triggers cell growth and differentiation [31]. Meanwhile, VEGF increases neurogenesis and promotes cell survival after traumatic brain injury *in vivo* by activating PI3K/Akt signal pathways [17]. Akt promotes neuronal differentiation of NPCs by regulating the assembly and activity of neural basic helix-loop-helix (bHLH) transcription factor complexes [32]. Signal transducer and activator of transcriptions (STATs), as cytoplasmic transcription factors, are activated at the plasma membrane by tyrosine phosphorylation before being translocated into the nucleus to activate target genes [33]. VEGF-induced reprogramming of epithelial progenitor cells (EPCs) could promote neurogenesis through modulating Flk1 expression and phosphorylation of Stat3 [34]. Moreover, Stat3 can be phosphorylated by p44/42MAPK on Ser-727 in response to growth factors to promote neuronal differentiation in embryonic stem cells [35], [36]. Thus, p44/42MAPK, Akt and Stat3 are potential target proteins in VEGF-mediated neuronal growth and differentiation.

Recent progress indicates that caveolin-1 (Cav-1), a 22 kDa protein located at plasma membrane invaginations, negatively controls VEGF signaling pathways. Over-expression of Cav-1 can inhibit flk1 and p44/42MAPK cascade and block VEGF-induced cell growth signals in endothelial cells [37]. Cav-1 is widely expressed in neuronal cell types and brain regions [38], [39]. Cav-1 decreases neurite outgrowth and branching, and reduces neurite density in differentiated PC 12 cells [40]. Cav-1 blocks the formation of neurites and phosphorylation of ERK in bFGF-treated N2a cells [41]. Our previous study revealed that Cav-1 was down-regulated in the core and penumbra of ischemic brains [42]. Cav-1 was found in NPCs [43]. Genetic ablation of Cav-1 increased NPCs proliferation in the subventricular zone (SVZ) of the adult mouse brain [44]. Whether Cav-1 regulates VEGF signaling pathway and neuronal differentiation of NPCs is still unknown. In the present study, we conducted a series of experiments to test the hypothesis that (1) Cav-1 can affect the differentiation of NPCs by inhibiting VEGF, p44/42 MAPK, PI3K/Akt and Stat3 signaling pathways in NPCs; (2) Hypoxia can down-regulate the expression of Cav-1 protein in NPCs, and the subsequently down-regulation of Cav-1 can activate p44/42 MAPK, PI3K/Akt and Stat3 signaling cascades in a VEGF-dependent pathway and subsequently promote neuronal differentiation of NPCs.

## Results

### Cav-1 knockout mice had enhanced neuronal differentiation of NPCs and VEGF expression in hippocampal dentate gyrus of brains

**Figure 1:**
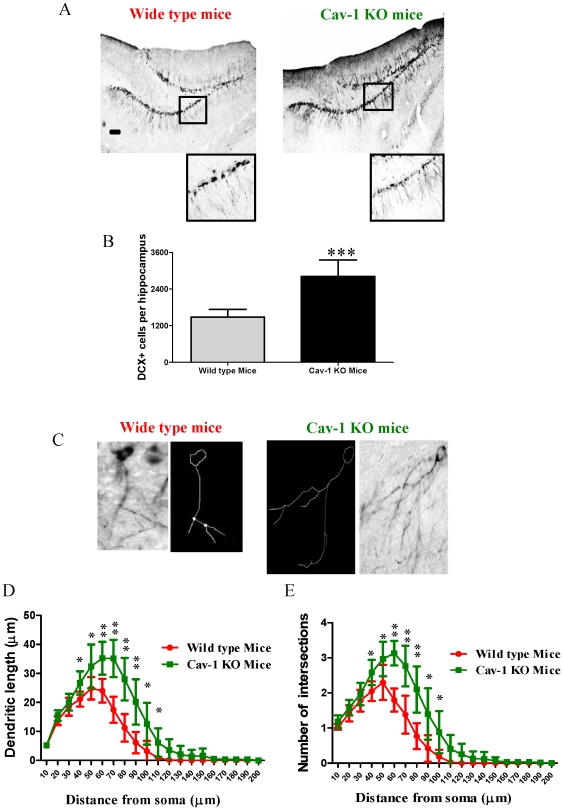
Neuronal regeneration in the granule cell layer of the hippocampal dentate gyrus of wild type and Cav-1 KO mice. **A.** Representative micrographs of the immature neuronal marker, doublecortin (DCX), in the granule cell layer of the hippocampal dentate gyrus of wild type mice and Cav-1 KO mice. Scale bar = 100 µm **B.** Histograms showing the quantification of DCX positive cells in wild type mice and Cav-1 KO mice (Mean ± S.D., n = 6). Wild type mice versus Cav-1 KO mice, *** p<0.001. **C.** Representative image and traces from Sholl analysis of DCX positive cells with tertiary branches in wild type mice and Cav-1 KO mice. **D.** Cav-1 KO mice had longer dendrites of DCX positive cells than wild type mice (Mean ± S.D., n = 6). The number of intersection/dendritic length represents the complexity of the dendrites. Wild type mice versus Cav-1 KO mice, *p<0.05, p<0.01; **E.** Cav-1 KO mice had higher numbers of intersections of DCX positive cells than wild type mice (Mean ± S.D., n = 6). Wild type mice versus Cav-1 KO mice, * p<0.05, p<0.01.

**Figure 2:**
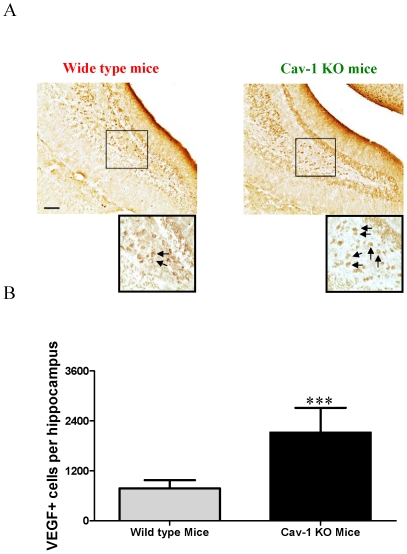
VEGF expression in the granule cell layer of the hippocampal dentate gyrus of wild type and Cav-1 KO mice. **A,** Representative micrographs of VEGF positive cells in the granule cell layer of the hippocampal dentate gyrus of wild type mice and Cav-1 KO mice. Arrows indicate the positive cells. Scale bar = 100 µm **B,** Histograms showing the quantification of VEGF positive cells in wild type mice and Cav-1 KO mice (Mean ± S.D., n = 6). Wild type mice versus Cav-1 KO mice, *** p<0.001.

In order to elucidate the role of Cav-1 in regulating neuronal development *in vivo*, both wild type C57BL/6J mice and Cav-1 knockout mice derived from C57BL/6J strain were used in this study, and we compared the rates of doublecortin (DCX) positive cells in the granule cell layer of the hippocampal dentate gyrus, one of the major areas of neurogenesis. DCX gene encodes a 40-kDa microtubule-associated protein, which is specifically expressed in neuronal precursors in the developing and adult CNS. During CNS development, the DCX expression is associated with the migration and differentiation of neuronal precursors [54], [55]. Thus DCX was used as a biomarker for detecting neuronal differentiation and development. As shown in Figure 1, Cav-1 knockout mice had more abundant DCX immunoreactive cells than wild type mice (about 1.9-fold increase) (Fig. 1A, 1B). Moreover, in the hippocampal dentate gyrus, Cav-1 knockout mice had higher numbers of DCX-immunoreactive cells with longer tertiary dendrites than wild type mice (Fig. 1C–1E). In the meantime, we investigated the expression of VEGF in the hippocampal dentate gyrus of wild type mice and Cav-1 knockout mice. As shown in Figure 2, the Cav-1 knockout mice had more abundant VEGF positive cells than wild type mice (about 2.7-fold increase). These results indicate that the genetic ablation of Cav-1 directly promotes the productions and maturations of newborn neurons and VEGF expression.

### Hypoxia-reoxygenation (HR) treatment down-regulated Cav-1 expression but enhanced neuronal differentiation in NPCs

**Figure 3:**
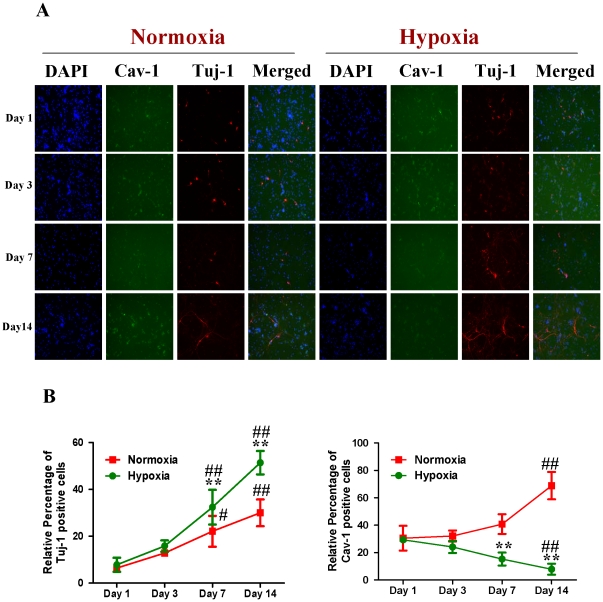
Dynamic changes of Cav-1 and differentiation of NPCs under normoxic and hypoxia-reoxygenation conditions. For hypoxia-reoxygenation (HR) treatment, NPCs were exposed 1% to O_2_ for 24 h and then switched to 21% O_2_ for 1, 3, 7, and 14 days, while for normoxic treatment, NPCs were consistently cultured under normoxia with 21% O_2_. Neuronal differentiation was identified by using Tublin β-III (Tuj-1). The fluorescent images were obtained with Carl Zeiss Axio Observer Z1 fluorescent imaging system. **A. Representative immunofluorescent imaging of Tuj-1 and Cav-1 in NPCs:** Red color: Tuj-1 staining; Green color: Cav-1 staining. Nuclear localization of Tuj-1 and Cav-1 was verified by co-localization with DAPI staining (blue color). **B. Statistical analysis on the relative percentage of Cav-1 and Tuj-1 positive cells in NPCs** (Mean ± S.D., n = 5). Hypoxia versus Normoxia at same time points, ** p<0.01; Observed day versus day 1 under the same oxygen condition, # p<0.05; ## p<0.01.

**Figure 4:**
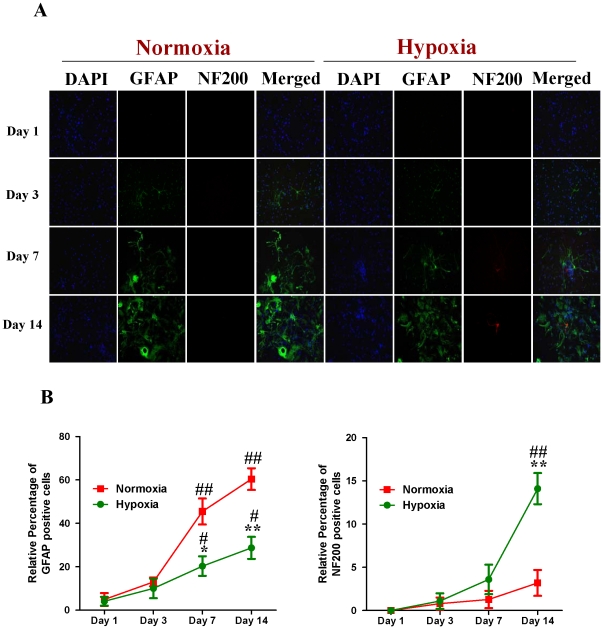
Neuronal and astroglial differentiation of NPCs under normoxic and hypoxia-reoxygenation conditions. For HR treatment, NPCs were exposed 1% to O_2_ for 24 h and then switched to 21% O_2_ for 1, 3, 7, and 14 days, while for normoxic treatment, NPCs were consistently cultured under normoxia with 21% O_2_. Neuronal differentiation was identified by using NF200 whereas glial differentiation was observed by detecting GFAP expression. The fluorescent images were obtained with Carl Zeiss Axio Observer Z1 fluorescent imaging system. **A. Representative immunofluorescent imaging of Neurofilament 200 (NF 200) and glial fibrillary acidic protein (GFAP) in NPCs.** Red color: NF200 staining; Green color: GFAP staining. Nuclear localizations of NF200 and GFAP were verified by co-localization with DAPI staining (blue color). **B. Statistical analysis on the relative percentage of GFAP and NF200 positive cells in NPCs** (Mean ± S.D., n = 6). Hypoxia versus normoxia at same time points, * p<0.05, ** p<0.01; Observed day versus day 1 under the same oxygen condition, # p<0.05, ## p<0.01.

**Figure 5:**
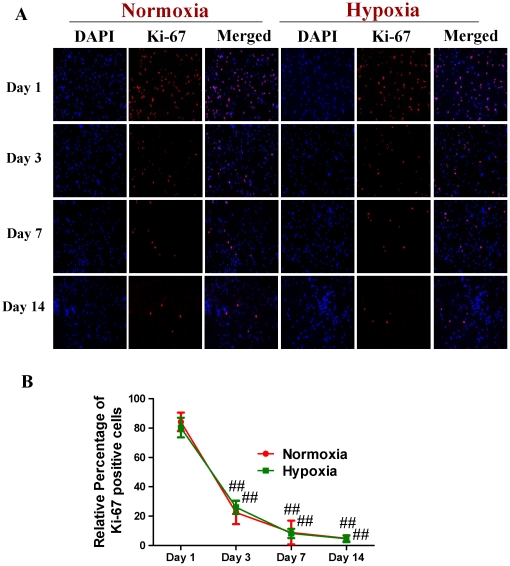
The proliferation of NPCs under normoxic and hypoxia-reoxygenation conditions. For HR treatment, NPCs were exposed 1% to O_2_ for 24 h and then switched to 21% O_2_ for 1, 3, 7, and 14 days, while for normoxic treatment, NPCs were consistently cultured under normoxia with 21% O_2_. **A. Representative immunofluorescent imaging of Ki-67 in NPCs.** Red color: Ki-67 staining; Nuclear localization of Ki-67 was verified by co-localization with DAPI staining (blue color). **B. Statistical analysis on the relative percentage of Ki-67 positive cells in NPCs** (Mean ± S.D., n = 6). Observed day versus day 1 under the same oxygen condition, ## p<0.01.

**Figure 6:**
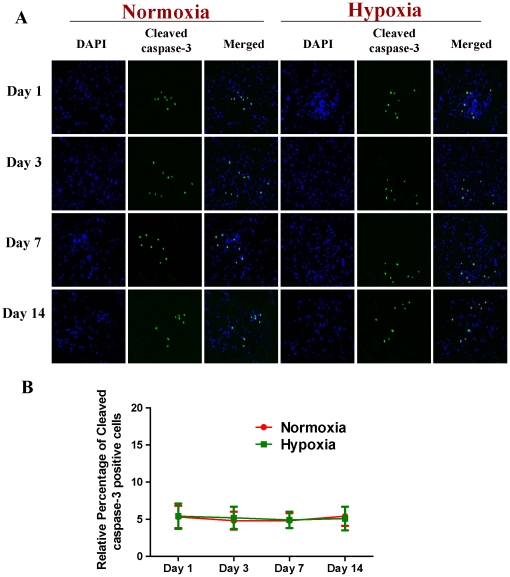
The apoptosis of NPCs under normoxic and hypoxia-reoxygenation conditions. For HR treatment, NPCs were exposed 1% to O_2_ for 24 h and then switched to 21% O_2_ for 1, 3, 7, and 14 days, while for normoxic treatment, NPCs were consistently cultured under normoxia with 21% O_2_. **A. Representative immunofluorescent imaging of Cleaved caspase-3 in NPCs.** Green color: Cleaved caspase-3 staining; Nuclear localization of Cleaved caspase-3 was verified by co-localization with DAPI staining (blue color). **B. Statistical analysis on the relative percentage of Cleaved caspase-3 positive cells in NPCs** (Mean ± S.D., n = 6).

**Figure 7:**
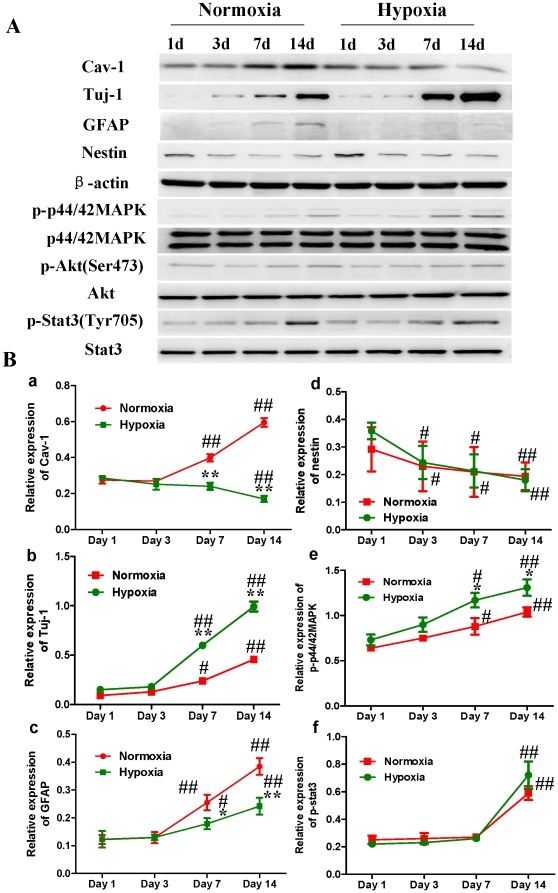
Western blot analysis on the expressions of Cav-1, Tuj-1, GFAP, nestin, p-p44/42MAPK, p-Akt and p-Stat3 proteins in NPCs under normoxic and hypoxia-reoxygenation conditions. For HR treatment, NPCs were exposed 1% to O_2_ for 24 h and then switched to 21% O_2_ for 1, 3, 7, and 14 days, while for normoxic treatment, NPCs consistently cultured under normoxia with 21% O_2_. **A. Representative immunoblot detections for the expressions of Cav-1, Tuj-1, GFAP, nestin, p-p44/42MAPK, p-Akt and p-Stat3.** Cell lysates were blotted with antibodies including Cav-1, Tuj-1, GFAP, nestin, p-p44/42 MAPK, p-Akt, and p-Stat3, in which β-actin, p44/42 MAPK, Akt and Stat3 were used as internal references, respectively. **B. Statistical analysis on the expressions of Cav-1, Tuj-1, GFAP, nestin, p-p44/42MAPK and p-Stat3** (Mean ± S.D., n = 3). Expressions of Cav-1, Tuj-1, GFAP and nestin were presented as fold activation of light units normalized to β-actin, whereas the phosphorylations of p44/42MAPK, Akt, and Stat3 were presented as the fold activations of light units normalized to p44/42MAPK, Akt and Stat3, respectively; Hypoxia versus normoxia at same time points, * p<0.05, ** p<0.01; Observed day versus day 1 under the same oxygenation condition, # p<0.05, ## p<0.01. Each sample was assayed at least 3 times.

Subsequently, we compared the expression of Cav-1 protein and neuronal differentiation of NPCs under normoxic and HR conditions. Tuj-1 is a commonly used neuronal biomarker. By co-immunostaining of Cav-1, Tuj-1 and DAPI, we investigated the dynamic changes of Cav-1 expression and neuronal differentiation in NPCs after the cells were exposed to hypoxia with 1% O_2_ for 24 h and switched to normoxia with 21% O_2_ for 1, 3, 7 and 14 days. As shown in Figure 3(A, B), the expression of Cav-1 was increased in normoxic NPCs but decreased in HR-treated NPCs. Simultaneously, HR-treated NPCs had higher level of Tuj-1 expression than normoxic NPCs. Subsequently, by co-immunostaining of a mature neuronal marker neurofilament 200 (NF200) and glial marker glial fibrillary acidic protein (GFAP), we further identified the formations of neurons and glial cells derived from NPCs, respectively. As shown in Figure 4 (A, B), HR treatment promoted the maturation of neuronal cells but induced a less intense increase in the formation of glial cells in comparison to the normoxic condition. In the meantime, compared with normoxic NPCs, HR-treated NPCs had no significant changes in both Ki-67 (Fig. 5A, B) and cleaved caspase-3 positive cells (Fig. 6A, B). Similarly, immunoblot analysis (Fig. 7A, B) revealed that the expression levels of Cav-1 protein in normoxic NPCs at 1, 3, 7 and 14 days were gradually increased but were significantly decreased in HR-treated NPCs at day 7 and day 14. The expression of Tuj-1 was markedly enhanced at day 7 and day 14 in both normoxic and HR-treated NPCs. HR treatment induced a relatively higher level of Tuj-1 expression than normoxic treatment at day 7 and day 14. Consistent with GFAP fluorescent staining, HR treatment induced a less intense increase in the expression of GFAP protein at day 7 and day 14 in comparison to the normoxic condition. There was no statistical change in the expression of nestin between normoxic NPCs and HR-treated NPCs. These results suggest that the down-regulation of Cav-1 protein might be associated with enhanced neuronal differentiation instead of promoting proliferation and inducing apoptosis in HR-treated NPCs.

### Hypoxia-reoxygenation (HR) treatment promoted VEGF expression, p44/42 MAPK phosphorylation and neuronal differentiation in NPCs

We next compared the phosphorylations of p44/42MAPK, Akt, Stat3 in normoxic and HR-treated NPCs (Fig. 7A, 7B, Fig. S1). Immunoblot analysis revealed that the phosphorylation of p44/42MAPK was up-regulated at day 7 and 14 in both normoxic and HR-treated NPCs. The HR-treated NPCs had a significant higher phosphorylation level of p44/42MAPK than normoxic NPCs. Meanwhile, the phosphorylation of Akt had no significant change in all time points whereas the phosphorylation of stat3 was significantly up-regulated at day 14 in both normoxic and HR-treated NPCs. Compared with normoxic NPCs, HR-treated NPCs had no effect on the phosphorylation of Stat3 in the current experimental condition.

**Figure 8:**
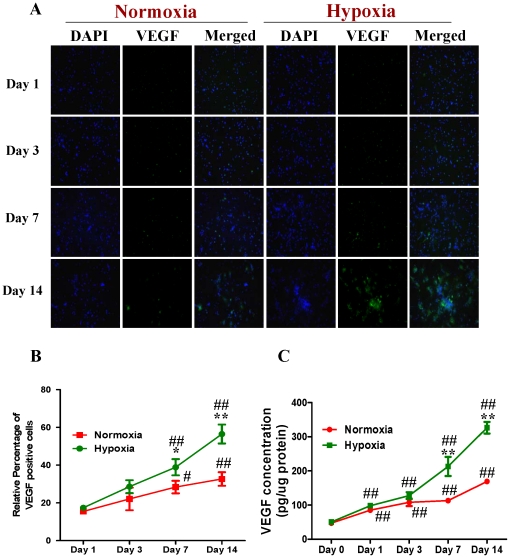
Expression of VEGF in NPCs treated with normoxia and hypoxia-reoxygenation. For HR treatment, NPCs were exposed 1% to O_2_ for 24 h and then switched to 21% O_2_ for 1, 3, 7, and 14 days, while for normoxic treatment, NPCs consistently cultured under normoxia with 21% O_2_. **A. Representative immunofluorescent imaging of VEGF expression in NPCs:** Green color: VEGF staining. Nuclear localization of VEGF was verified by co-localization with DAPI staining (blue color). **B. Statistical analysis on the relative percentage of VEGF positive cells in NPCs** (Mean ± S.D., n = 6). Hypoxia versus normoxia at same time points, * p<0.05, ** p<0.01; Observed day versus day 1 under the same oxygenation condition, # p<0.05, ## p<0.01. **C. ELISA detection of VEGF concentrations in culture media from normoxic and hypoxic NPCs.** Cell culture supernatants were collected from the normoxic and hypoxic NPCs at 0, 1, 3, 7 and 14 days. VEGF was detected with ELISA immunoassay kit (R&D systems) according to manufacturer's protocol (Mean ± S.D., n = 3). Hypoxia versus normoxia at same time points, ** p<0.01; Observed day versus day 0 under the same oxygenation condition, ## p<0.01. Each sample was measured in duplicate.

By co-immunostaining of VEGF and DAPI, we investigated the intracellular levels of VEGF in both normoxic and HR-treated NPCs with fluorescent microscopy. Figure 8 showed that HR treatment promoted VEGF expression in NPCs (Fig. 8A, 8B). We subsequently determined the levels of secreted VEGF in the differentiation medium. Consistently, the ELISA experiments showed that the level of VEGF was increased in both normoxic and HR-treated NPCs. The HR-treated NPCs had a remarkable higher level of VEGF than normoxic NPCs at day 7 and day 14 (Fig. 8C). These results suggest that hypoxia-reoxygenation treatment can induce the up-regulation in the phosphorylation of p44/42MAPK and VEGF expression, and promote neuronal differentiation of NPCs.

### Cav-1 manipulations regulate neuronal differentiation and expressions of VEGF, flk1, phosphorylations of p44/42MAPK, Akt and Stat3 in normoxic NPCs

**Figure 9:**
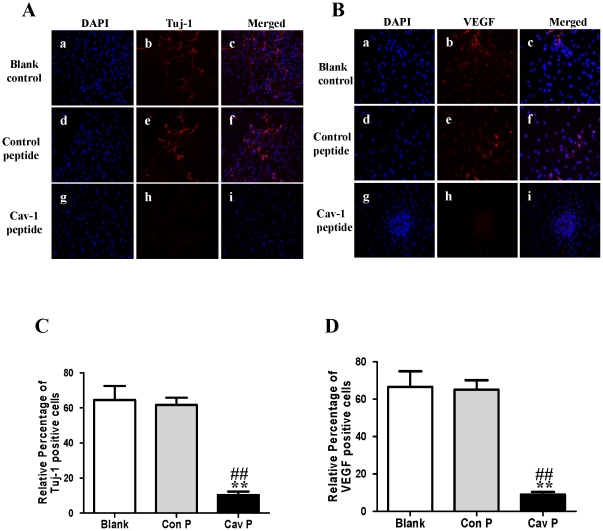
Effects of Cav-1 scaffolding domain peptide on the expressions of Tuj-1 and VEGF in NPCs under normoxic condition. Cells were consistently incubated in a standard incubator with humidified 21% O_2_ plus 5% CO_2_ balanced with N_2_ for 14 days. Cells were cultured with fresh medium containing a synthetic cell-permeable peptide encoding Cav-1 scaffolding domain (amino acids 82 to 101, DGIWKASFTTETVTKYWFYR) or a Cav-1 scrambled control peptide (WGIDKAFFTTSTVTYKWFRY) with Antennapedia internalization sequence (RQIKIWFQNRRMKWKK) at a final concentration of 4 µM. The medium was changed every two days and lasted for 14 days. **A–B. Representative immunofluorescent imaging of Tuj-1 and VEGF in NPCs at day 14.** Red color: Tuj-1 and VEGF staining; Tuj-1 and VEGF staining (red color) were identified in blank control group (**a**, **b**, **c**), control peptide group (**d**, **e**, **f**) and Cav-1 peptide group (**g**, **h**, **i**). Nuclear localizations of Tuj-1 and VEGF were verified by co-localization with DAPI staining (blue color). **C–D. Statistical analysis on the relative percentage of Tuj-1 and VEGF positive cells in NPCs** (Mean ± S.D., n = 6). Blank, blank control group; Con P, Cav-1 scrambled control peptide group; Cav P, Cav-1 scaffolding domain peptide group. Cav P versus blank, ** p<0.01; Cav P versus Con P, ## p<0.01.

**Figure 10:**
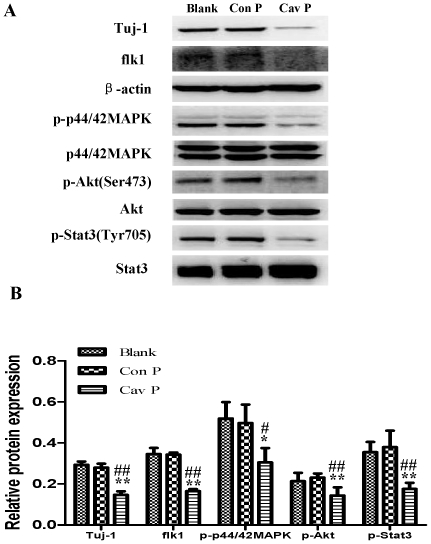
Effects of Cav-1 scaffolding domain peptide on the expressions of Tuj-1, flk1, p-p44/42MAPK, p-Akt and p-Stat3 proteins in NPCs under normoxic condition. Similar protocols as Figure 7 were used. Expressions of Tuj-1, flk1, p-p44/42MAPK, p-Akt and p-Stat3 proteins in NPCs at day 14 were analyzed with western blot analysis. Cell lysates were blotted with the antibodies of Tuj-1, flk1, p-p44/42MAPK, p-Akt, and p-Stat3, in which β-actin, p44/42MAPK, Akt and Stat3 were used as internal references, respectively. **A. Representative immunoblot results of Tuj-1, flk1, p-p44/42MAPK, p-Akt and p-Stat3.** Blank, blank control group; Con P, Cav-1 scrambled control peptide group; Cav P, Cav-1 scaffolding domain peptide group. **B. Statistical analysis on the expressions of Tuj-1, flk1, p-p44/42MAPK, p-Akt and p-Stat3** (Mean ± S.D., n = 3). The expressions of Tuj-1 and flk1 were presented as fold activation of light units normalized to β-actin, whereas the phosphorylations of p44/42MAPK, Akt, and Stat3 were presented as fold activations of light units normalized to p44/42MAPK, Akt and Stat3, respectively. Cav P versus blank, * p<0.05, ** p<0.01; Cav P versus Con P, # p<0.05, ## p<0.01; Each sample was assayed at least 3 times.

**Figure 11:**
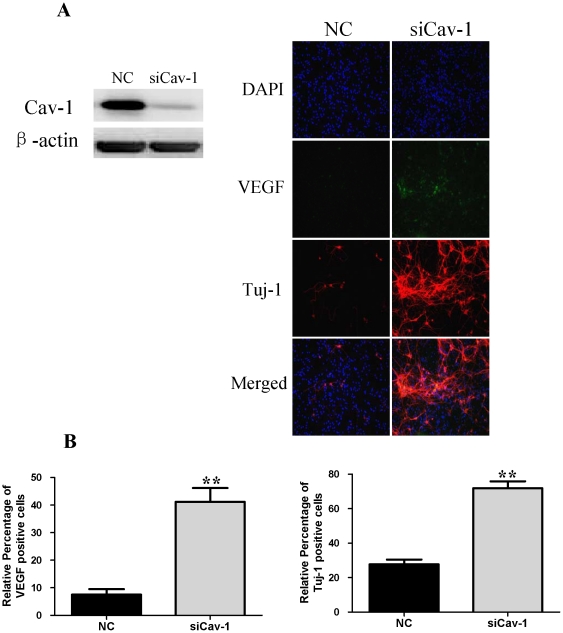
Effects of siRNA Cav-1 specific knockdown on the expressions of Tuj-1, VEGF in NPCs with or without V1 treatment under normoxic condition. A short interfering RNA transfection was used to knock down the expression of Cav-1 in NPCs. NPCs were transfected with Cav-1 Stealth™ RNAi. All data were obtained at day 14. **A. Representative results of the expressions of Cav-1, Tuj-1 and VEGF.** Left, the expression of Cav-1 was successfully knocked down by the Cav-1 RNAi; Right, immunofluorescent imaging of VEGF and Tuj-1, Green color: VEGF staining. Red color: Tuj-1 staining. Nuclear localization of VEGF and Tuj-1 was verified by co-localization with DAPI staining (blue color). NC, negative control group; siCav-1, Cav-1 RNA silencing group; **B. Statistical analysis on the relative percentage of VEGF and Tuj-1 positive cells in NPCs** (Mean ± S.D., n = 6). siCav-1 versus NC, **p<0.01.

**Figure 12:**
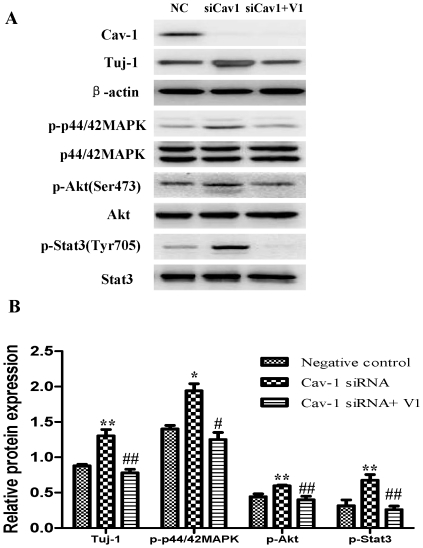
Effects of siRNA Cav-1 specific knockdown on the expressions of Tuj-1, p-p44/42MAPK, p-Akt and p-Stat3 in NPCs with or without V1 treatment under normoxic condition. A short interfering RNA transfection was used to knock down the expression of Cav-1 in NPCs. NPCs were transfected with Cav-1 Stealth™ RNAi. All data were obtained at day 14. **A. Representative immunoblot results for the expressions of Tuj-1, p-p44/42MAPK, p-Akt and p-Stat3 in NPCs treated by Cav-1 specific knockdown with or without V1 treatment.** VEGF inhibitor V1 (12 µM) was used to treat NPCs prior to transfection of Cav-1 Stealth™ RNAi. Cell lysates were blotted with the antibodies for Tuj-1, p-p44/42 MAPK, p-Akt, and p-Stat3, in which β-actin, p44/42MAPK, Akt and Stat3 were used as internal references, respectively. NC, negative control group; siCav-1, Cav-1 RNA silencing group; siCav-1+V1: Cav-1 RNA silencing+V1 group; **B. Statistical analysis on the expressions of Tuj-1, flk1, p-p44/42MAPK, p-Akt and p-Stat3** (Mean ± S.D., n = 3). The expression of Tuj-1 was presented as fold activation of light units normalized to β-actin, whereas the phosphorylations of p44/42MAPK, Akt, and Stat3 were presented as the fold activations of light units normalized to p44/42MAPK, Akt and Stat3, respectively. Cav-1 siRNA versus negative control, * p<0.05, ** p<0.01; Cav-1 siRNA+V1 versus Cav-1 siRNA, # p<0.05, ## p<0.01; Each sample was assayed at least 3 times.

To explore the impact of Cav-1 on the VEGF expression, phosphorylations of p44/42MAPK, Akt, Stat3 and neuronal differentiation, we artificially manipulated the level of Cav-1 and determined the expression of Tuj-1 in NPCs under normoxic condition. The cells were treated with a Cav-1 scaffolding domain peptide (Cav-1 peptide) or a Cav-1 scrambled control peptide (control peptide). Since day 14 had remarkably the enhanced neural differentiation and the decreased Cav-1 level in HR-treated NPCs, we particularly selected day 14 for the experiments. The treatment of Cav-1 peptide, instead of control peptide, remarkably inhibited neuronal differentiation (Fig. 9A, Fig. 9C, Fig. 10) and down-regulated the expressions of VEGF and its receptor flk1 protein (Fig. 9B, Fig. 9D, Fig. 10). In addition, Cav-1 peptide remarkably down-regulated the phosphorylations of p44/42MAPK, Akt, and Stat3 in NPCs (Fig. 10, Fig.S2). We further investigated the relationship between Cav-1 expression and neuronal differentiation in NPCs by knocking down Cav-1 with an RNA silencing approach. The RNAi treatment resulted in about 65% inhibition in the expression of Cav-1 (Fig. 11A). By co-immunostaining of VEGF, Tuj-1 and DAPI, we found that the knockdown of Cav-1 significantly up-regulated the expressions of VEGF and Tuj-1 (Fig. 11A, 11B). Immunoblot analysis further confirmed that Cav-1 RNAi treatment not only promoted VEGF expression and neuronal differentiation of NPCs but also up-regulated the phosphorylations of p44/42MAPK, Akt, and Stat3 in normoxic NPCs (Fig. 12A, 12B, Fig. S3).

**Figure 13:**
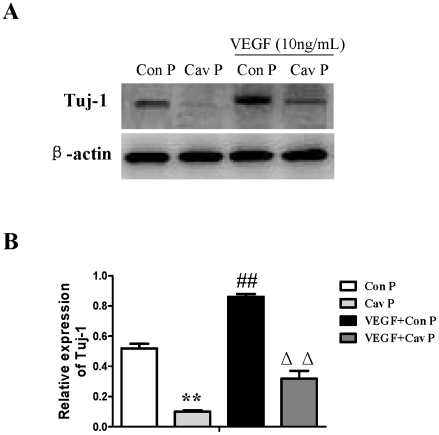
Effects of Cav-1 scaffolding domain peptide on the expression of Tuj-1 in NPCs treated with VEGF under normoxic condition. Cells were consistently incubated with fresh medium containing VEGF (10 ng/ml) and a Cav-1 scaffolding domain peptide (4 µM) or a Cav-1 scrambled control peptide (4 µM) with Antennapedia internalization sequence in a standard incubator at humidified 21% O_2_ plus 5% CO_2_ balanced with N_2_. The medium was changed every 2 days and cells were cultured for 14 days. All data were obtained from the samples at day 14. **A. Representative immunoblot results for the expressions of Tuj-1.** Cell lysates were blotted with the Tuj-1 antibody, in which β-actin was used as internal reference. Con P, Cav-1 scrambled control peptide; Cav P, Cav-1 scaffolding domain peptide. **B. Statistical analysis on the expression of Tuj-1.** (Mean ± S.D., n = 3). Cav P versus Con P, VEGF+Con P and VEGF+Cav P, ** p<0.01; VEGF+Con P versus Con P, Cav P and VEGF+Cav P, ## p<0.01; VEGF+Cav P versus VEGF +Con P, Con P and Cav P, ΔΔ p<0.01.

We then investigated the roles of VEGF signaling in the regulation of neuronal differentiation induced by Cav-1. To achieve this goal, we observed the effects of VEGF specific inhibitor (V1) on Tuj-1 expression in the Cav-1 RNAi-treated NPCs. V1 is a dimerized peptide derived from the third Ig-like domain of the human vascular endothelial growth factor receptor-2 (KDR/Flk1) comprising residues 247–261 [56], [57]. V1 can bind to VEGF and block its interaction with VEGFR-2 and the autophosphorylation of VEGFR-2 [58], [59]. As expected, VEGF inhibitor abolished the Cav-1 RNAi-induced up-regulations in the phosphorylations of p44/42MAPK, Akt, Stat3 and the neuronal differentiation of NPCs (Fig. 12A, 12B, Fig. S3). On the other hand, we also investigated the effects of Cav-1 scaffolding domain peptide on VEGF-induced neuronal differentiation. The results revealed that VEGF treatment promoted neuronal differentiation of NPCs as showed by Tuj-1 expression, but the neurogenesis-promoting effects of VEGF were remarkably inhibited by Cav-1 peptide treatment (Fig. 13). These results provide direct evidence that Cav-1 can inhibit neuronal differentiation of NPCs via the down-regulations of VEGF/flk1 and its downstream signaling molecules including p44/42MAPK, Akt and Stat3.

### Cav-1 manipulations affect neuronal differentiation and expressions of VEGF, flk1, phosphorylations of p44/42MAPK, Akt and Stat3 in HR-treated NPCs

**Figure 14:**
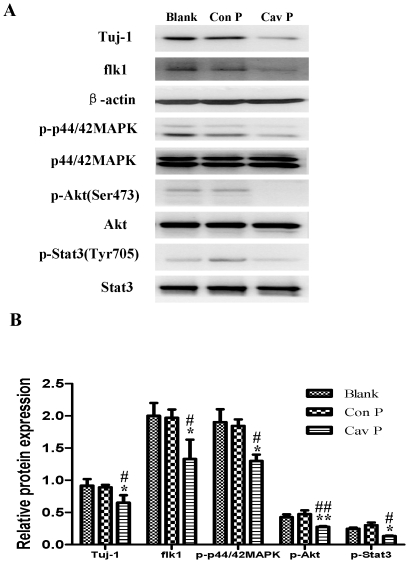
Effects of Cav-1 scaffolding domain peptide on the expressions of Tuj-1, flk1, p-p44/42MAPK, p-Akt and p-Stat3 proteins in NPCs under hypoxia-reoxygenation condition. NPCs were cultured with fresh medium containing a synthetic cell-permeable Cav-1 scaffolding domain peptide (4 µM) or a Cav-1 scrambled control peptide (4 µM). For hypoxia-reoxygenation treatment, cells were exposed 1% O_2_ for 24 h and then switched to normoxia with fresh medium for 14 days. All data were obtained from the samples at day 14. **A. Representative results of immunoblot analysis for the expressions of Tuj-1, flk1, p-p44/42MAPK, p-Akt and p-Stat3.** Cell lysates were blotted with the antibodies for Tuj-1, flk1 p-p44/42 MAPK, p-Akt, and p-Stat3, in which β-actin, p44/42MAPK, Akt and Stat3 were used as internal references, respectively. Blank, blank control group; Con P, Cav-1 scrambled control peptide group; Cav P, Cav-1 scaffolding domain peptide group. **B. Statistical analysis on the expressions of Tuj-1, flk1, p-p44/42MAPK, p-Akt and p-Stat3** (Mean ± S.D., n = 3). The expressions of Tuj-1 and flk1 were presented as fold activation of light units normalized to β-actin, whereas the phosphorylations of p44/42MAPK, Akt, and Stat3 were presented as the fold activations of light units normalized to p44/42MAPK, Akt and Stat3, respectively. Cav P versus Blank, * p<0.05, ** p<0.01; Cav P versus Con P, # p<0.05, ## p<0.01. Each sample was assayed at least 3 times.

**Figure 15:**
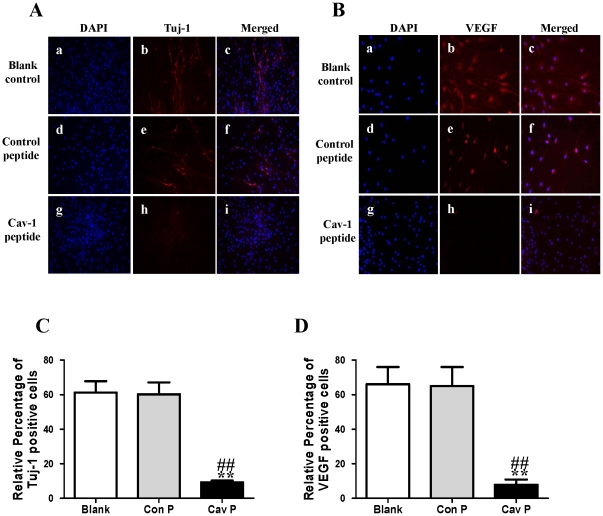
Effects of Cav-1 scaffolding domain peptide on the expressions of Tuj-1 and VEGF protein in NPCs under hypoxia-reoxygenation condition. Similar experimental protocols as Figure 9 were used in the experiments and data were obtained from the samples at day 14. **A–B: Representative immunofluorescent imaging of Tuj-1 and VEGF in the hypoxic NPCs at day 14.** Red color: Tuj-1 and VEGF staining; Tuj-1 and VEGF staining (red color) were identified in blank control group (**a**, **b**, **c**), control peptide group (**d**, **e**, **f**) and Cav-1 peptide group (**g**, **h**, **i**). Nuclear localizations of Tuj-1 and VEGF were verified by co-localization with DAPI staining (blue color). **C–D. Statistical analysis on the relative percentage of Tuj-1 and VEGF positive cells in NPCs** (Mean ± S.D., n = 6). Blank, blank control group; Con P, Cav-1 scrambled control peptide; Cav P, Cav-1 scaffolding domain peptide group. Cav P versus blank, ** p<0.01; Cav P versus Con P, ## p<0.01.

**Figure 16:**
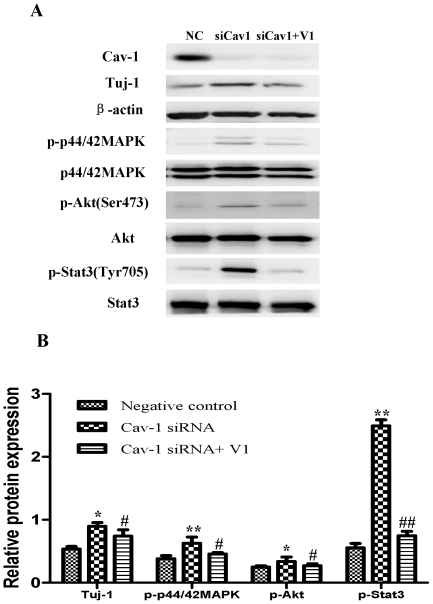
Effects of siRNA Cav-1 specific knockdown on the expressions of Tuj-1, p-p44/42MAPK, p-Akt and p-Stat3 proteins in NPCs under hypoxia-reoxygenation conditions with or without V1 treatment. For HR treatment, NPCs were exposed 1% O_2_ for 24 h and then switched to normoxia with fresh medium for 14 days. A short interfering RNA transfection was used to knock down the expression of Cav-1 in NPCs. NPCs were transfected with Cav-1 Stealth™ RNAi. V1 (12 µM) was used to treat NPCs prior to transfection of Cav-1 Stealth™ RNAi. All data were obtained at day 14. **A. Representative results of immunoblot analysis for the expressions of Tuj-1, p-p44/42MAPK, p-Akt, p-Stat3.** Cell lysates were blotted with the antibodies for Tuj-1, p-p44/42MAPK, p-Akt and p-Stat3 in which the antibodies for β-actin, p44/42 MAPK, Akt and Stat3 were used as internal references, respectively. NC, negative control group; siCav-1, Cav-1 RNA silencing group; siCav-1+V1: Cav-1 RNA silencing+V1 group; **B. Statistical analysis on the expressions of Tuj-1, flk1, p-p44/42MAPK, p-Akt and p-Stat3** (Mean ± S.D., n = 3). The expression of Tuj-1 was presented as fold activation of light units normalized to β-actin, whereas the phosphorylations of p44/42MAPK, Akt, and Stat3 were presented as the fold activations of light units normalized to p44/42 MAPK, Akt and Stat3, respectively. Cav-1 siRNA versus negative control, * p<0.05, ** p<0.01; Cav-1 siRNA+V1 versus Cav-1 siRNA, # p<0.05, ## p<0.01. Each sample was assayed at least 3 times.

Finally, by using similar approaches to normoxic experiments, we further investigated the roles of Cav-1 in modulating neuronal differentiation in HR-treated NPCs. NPCs were exposed to hypoxia with 1% O_2_ for 24 h and then changed to normoxic condition with 21% O_2_ for 1, 3, 7 and 14 days. Prior to hypoxic treatment, the NPCs were pretreated with Cav-1 scaffolding domain peptide or Cav-1 scrambled control peptide and the cells were continuously incubated with the peptides for 14 days. As showed in Figure 14 and 15, the treatment of Cav-1 peptide markedly down-regulated the expressions of VEGF and flk1, phosphorylations of p44/42MAPK, Akt, Stat3, and inhibited neuronal differentiation in HR-treated NPCs. In contrast, the treatment of Cav-1 RNAi significantly up-regulated the phosphorylations of p44/42MAPK, Akt, Stat3 and promoted neuronal differentiation, whereas the co-treatment of V1 blocked the effects of RNAi in the transient hypoxic NPCs (Fig. 16). In particular, Cav-1 RNAi led to more than 3-fold increase in the phosphorylation of Stat3, but this increase was completely abolished by co-treatment of V1. Taken together, these results strongly suggest that hypoxia-induced down-regulation of Cav-1 expression contributes to the enhanced neuronal differentiation of NPCs via up-regulation of VEGF signaling and phosphorylations of p44/42MAPK, Akt, Stat3.

## Discussion

In the present study, we report for the first time that: (1) Cav-1 knockout mice had more abundant newborn neurons and higher VEGF expression than wild type mice; (2) Cav-1 inhibited neuronal differentiation of NPCs via the down-regulation of VEGF signaling and the inhibition of p-p44/42MAPK, p-Akt and p-Stat3; (3) Hypoxia down-regulated the expression of Cav-1, up-regulated VEGF and p-p44/42MAPK, subsequently promoting neuronal differentiation of NPCs. Our results suggest that the hypoxia-induced down-regulation of Cav-1 and subsequent activation of VEGF/flk1 and p44/42MAPK signaling pathways contribute to neuronal differentiation of neural progenitor cells.

Cav-1 functions as a negative regulator of tissue or cell regeneration. Previous study indicate that the down-regulation of Cav-1 can activate ERK and p44/42MAPK signaling pathway and affect proliferation and migration of myogenic precursor cells (MPCs) toward the wounded area [60]. Genetic ablation of Cav-1 increases NPCs proliferation in the subventricular zone (SVZ) of the adult mouse brain [44]. Herein, we found that Cav-1 knockout mice had more abundant DCX and VEGF positive staining cells in the hippocampal dentate gyrus than wild type mice, and that the loss of Cav-1 was associated with the enhanced VEGF expression and neuronal differentiation of NPCs. The results provide a clue that Cav-1 might play a role in regulating VEGF signaling and neuronal differentiations.

VEGF and its receptor flk1 can improve the extension of neural stem/progenitor cells [22], [61]. Flk1 receptor tyrosine kinase is the major mediator of VEGF-dependent signaling. The induction of VEGF/Flk1 stimulates the binding, phosphorylation and activations of multiple downstream signal molecules such as MAPK, PKC, PI3K-Akt, etc [62], [63], [64], [65]. We found that hypoxia-reoxygenation treatment remarkably down-regulated the expression of Cav-1, up-regulated the expression of VEGF and phosphorylation of p44/42MAPK, and increased the expression of Tuj-1 in NPCs. One may argue that the potential effects of hypoxia-reoxygenation on the proliferation and apoptosis of NPCs might affect the experimental results. To address this, we stained Ki-67 and Cleaved caspase-3 positive cells and measured the expression of nestin in both normoxic and HR-treated NPCs. Our results revealed that hypoxia-oxygenation treatment did not change the numbers of Ki-67 positive and Cleaved caspase-3 positive cells, and nestin expression. Those results suggest that hypoxia-reoxygenation treatment can inhibit Cav-1, up-regulate VEGF and p44/42MAPK signaling, and promote neuronal differentiation of NPCs.

Cav-1 can serve as a scaffold for a variety of signaling complexes. The presence of a Cav-1 domain corresponding to amino acids 82–101 can interact with consensus motifs present in VEGF and its receptor flk1 [66]. To address the intrinsic relationship between the down-regulation of Cav-1 and the differentiation of NPCs, we chose to use Cav-1 scaffolding domain peptide and Cav-1 RNAi in the normoxic and hypoxic experiments. Cav-1 scaffolding domain peptide remarkably inhibited the expressions of VEGF, flk1, phosphorylation of p44/42MAPK and neuronal differentiation of NPCs. Cav-1 scaffolding domain peptide directly abolished the VEGF-induced neuronal differentiation. On the other hand, Cav-1 RNAi promoted the expressions of VEGF, flk1 and the phosphorylation of p44/42MAPK and neuronal differentiation NPCs under both normoxic and hypoxic conditions. Interestingly, VEGF inhibitor V1 abolished the Cav-1 RNAi-induced up-regulations in the phosphorylations of p44/42MAPK, Akt, Stat3 and the neuronal differentiation of NPCs. These results indicate that Cav-1 specially inhibits neuronal differentiation of NPCs through modulating VEGF/flk1 expressions and phosphorylation of p44/42MAPK. There is an avenue to mention that neuronal differentiation is complex and multiple signal pathways involve in the process. The down-regulation of VEGF/flk1 could be due to the direct effects of Cav-1 or indirect mechanisms via regulating other molecular cascades. For example, NO is known to promote the expression of VEGF whereas Cav-1 can inhibit endothelial nitric oxide synthase (eNOS) activity and decrease NO release [67], [68]. Thus, down-regulation of VEGF could be explained by the indirect roles of Cav-1 on NO production. In addition, our recent study revealed that Cav-1 peptide could regulate Notch signaling in NPCs [69]. Notch-Hes1 served as a convergent signaling node within early retinal progenitor cells to integrate cell-extrinsic cues such as VEGF and sonic hedgehog (SHH), to regulate cell proliferation and neuronal specification [18]. The interaction of Notch and VEGF might contribute to the molecular regulations of Cav-1 on VEGF signaling and neuronal differentiation. Therefore, we remark that Cav-1 plays a crucial role in inhibiting VEGF signaling and neuronal differentiation of NPCs. The down-regulation of Cav-1 contributes to the enhanced VEGF/flk1 expressions and p44/42MAPK phosphorylation and neurogenesis in hypoxia-reoxygenation-treated NPCs.

It is well known that Stat3 and Akt play important roles in growth factors-induced neuronal differentiation [70], [71]. Here, we found that the phosphorylations of Akt and Stat3 were down-regulated by Cav-1 peptide but up-regulated by Cav-1 RNAi in normoxic and hypoxic NPCs, suggesting that Cav-1 can inhibit activities of Akt and Stat3. However, exposure to 1% O_2_ did not induce a significant difference in the phosphorylations of Akt and Stat3 although hypoxic treatment induced the phosphorylation of p44/42MAPK. One feasible explanation could be that, although hypoxic treatment down-regulated Cav-1, the down-regulation of Cav-1 did not reach a point where phosphorylations of Akt and Stat3 were enhanced in NPCs. Moreover, we found that VEGF inhibitor (V1) completely abolished Cav-1 RNAi induced up-regulation in the phosphorylations of Akt and Stat3 in normoxic and hypoxic NPCs. As different phosphorylation sites may carry different functions, we also measured the effect of Cav-1 on the phosphorylations of Stat3 at the sites of Tyr 705 and Ser 727. Data showed that Cav-1 could negatively modulate phosphorylation of Stat3 at both serine and tyrosine sites. Especially, VEGF inhibitor abolished the enhanced phosphorylation of Stat3 at both serine and tyrosine sites in the Cav-1 RNAi-treated NPCs. These results imply that the Cav-1-induced inhibition of Akt and Stat3 signaling are VEGF dependent.

In conclusion, we report that Cav-1 can inhibit VEGF and its downstream p44/42 MAPK, Akt and Stat3 signaling pathways and modulate neuronal differentiation. The down-regulation of Cav-1 gene expression induced by hypoxia could improve neuronal differentiation of NPCs. Our data provide a potential explanation why hypoxia could enhance neuronal differentiation. These findings represent an important advance in understanding the signal pathways in the modulation of neurogenesis. Thus, Cav-1 could be developed into a novel therapeutic target protein for promoting neurogenesis for the treatment of stroke and neurodegenerative diseases in the future.

## Materials and Methods

### Animals

Cav-1 knockout mice (Cav-1 KO; Cav1 tm1Mls/J, Jackson Laboratory, Bar harbor, ME, USA) from heterozygote breedings were genotyped and used for the experiments. Wild type C57BL/6J mice and Sprague-Dawley (SD) rats were obtained from Laboratory Animal Unit at the University of Hong Kong. The experimental protocol was approved by the institutional Animal Care and Ethical Committee at the University of Hong Kong (Approved No. 1479-07, 1844-09). Every effort was made to minimize the number of animals used and their suffering. The animals were maintained at a controlled temperature (20±2°C) and group-housed (12-h light/dark cycle) with access to food and water *ad libitum*.

### Tissue processing and Immunohistochemistry

Wild-type and Cav-1 knockout (KO) mice at the age of 9 week old were deeply anesthetized with a ketamine/xylazine mixture solution, and then perfused transcardially with 0.05 M sodium phosphate-buffered saline (pH 7.4), followed by 4% paraformaldehyde in 0.05 M sodium phosphate-buffered saline (pH 7.4). Brains were removed and post-fixed overnight at 4°C. The brain tissues were then cryoprotected via 24 h immersion in 30% sucrose and cut into 40 µm sagittal sections. The brain sections were blocked with a solution containing 0.01 M PBS, 0.1% Triton X-100 and 5% normal goat serum solution for 30 min, and subsequently incubated overnight with rabbit anti-doublecortin (1∶500, DCX, Cell signal) and rabbit anti-VEGF (1∶50, Santa Cruz), respectively diluted in blocking solution. Sections were then washed extensively and incubated with secondary antibody (1∶200) and avidin-biotin complex using the Elite vectastain kit (Vector Laboratories, Burlingame, CA, USA). Chromogen reactions were performed with diaminobenzidine (DAB; sigma) and 0.1% H_2_O_2_ for 10 min. The sections were cover-slipped with Canada balsam (Sigma). Negative control groups were simultaneously performed by omitting primary antibodies to exclude the presence of non-specific staining.

To determine the number of DCX-positive cells in the hippocampus, every 12th sections (480 µm intervals) were selected from one cerebral hemisphere in each animal and processed for immunohistochemistry. The DCX-positive cells in the granule cell layer of the hippocampal dentate gyrus were manually counted using Stereo Investigator software (Version 8.11, Micro-Bright Field) with an automated stage by an experimenter blinded to the identities of animals. The amount of cells counted was then extrapolated to get an approximated value for whole brain. Dendritic complexity of DCX positive cells including dendritic length and number of intersections (branch points) in the granule cells layer of the hippocampal dentate gyrus were analyzed and calculated using Neurolucida software and NeuroExplorer software (Micro-Bright Field Biotechnology) [45], [46]. Dendritic length means the total dendritic length between the starting and ending radius [47]. Intersections represent total number of intersections between the specific concentric circles and the dendrites. A higher value in dendritic length or intersections represents a neuron with more complex dendritic branches. For the quantification of VEGF expression, six VEGF-immunostained coronal sections at 480 µm intervals were analyzed from each brain. Eight fields of view in each section were digitized under a 40× objective (Olympus BX51). The images obtained were analyzed by mean of Image J software (NIH, USA) [48], [49] and data were presented as the total numbers of VEGF immunoreactive cells within each field.

### Preparation of Fetal NPCs

NPCs were prepared from the cortex of embryonic E15-16 SD rats as previously described [50], [51]. Briefly, embryonic rats were deeply anesthetized with a ketamine/xylazine mixture solution, and then rat embryos were dissected and put into PBS containing 100 U/ml penicillin/streptomycin. The telencephalons were isolated. Fresh cerebrums were mechanically disrupted into single cells by filtering through a nylon mesh of 70 µm. After centrifugation at 1000×g for 5 min at 4°C, cells were re-suspended in serum-free Dulbecco's modified Eagle's medium/Ham's F12 medium (DMEM/F12; Invitrogen). Dissociated cells were seeded at a density of 1×10^5^cells/ml in DMEM/F12 replenished with 2% B27 (Invitrogen), recombinant human basic fibroblast growth factor (bFGF 10 ng/ml; Sigma), and epidermal growth factor (EGF 10 ng/ml; Sigma). Cells were incubated in a humidified atmosphere containing 5% CO_2_ at 37°C. Fresh culture medium containing the same concentration of trophic factors was added twice weekly. The purity of NPCs was verified to be >90% with nestin staining. Spheres appeared within a few days and were grown for 3–5 days before collecting them for passage. After one passage, cells were used for the experiments.

### Hypoxia/reoxygenation treatment

Isolated NPCs were re-suspended and cultured in Neurobasal medium containing 2% B27 and 0.2 mM L-glutamine, as in a multiple-well plate and then transferred into a modular incubator chamber. For normoxic treatment, NPCs were consistently cultured under normoxia with 21% O_2_. For hypoxia-reoxygenation treatment, NPCs were consistently incubated with 1% O_2_ plus 5% CO_2_ balanced with N_2_ for 24 h. After hypoxic treatment, the cells were returned to a standard incubator with the normoxic condition of humidified 95% air/5% CO_2_. During the experiments, the O_2_ concentration was monitored with PA-10A paramagnetic O_2_ analyzer (sable systems international). According to the designed experimental plans, we collected the cell samples at day 1, 3, 7 and 14 after the cells were exposed to 24 hours of normoxic or hypoxic treatments. Day 0 was defined as the onset after cells were exposed for 24 hours of normoxic or hypoxic treatments.

### Manipulations of Cav-1 and VEGF

To elucidate the effects of Cav-1 in regulating neuronal differentiation, a synthetic cell-permeable peptide encoding Cav-1 scaffolding domain (amino acids 82 to 101, DGIWKASFTTETVTKYWFYR) or the Cav-1 scrambled control peptide (WGIDKAFFTTSTVTYKWFRY) with Antennapedia internalization sequence (RQIKIWFQNRRMKWKK) was custom synthesized by SBS Biotech Co. as previously described [52]. A 10-mM stock solution was prepared with 100% DMSO and 4 µM of peptides was diluted in cultured medium. Fresh medium containing Cav-1 scaffolding domain peptide or Cav-1 scrambled control peptide was used to treat NPCs for 14 days. In a parallel group, fresh medium containing VEGF (Peprotech, 10 ng/ml) with Cav-1 scaffolding domain peptide or Cav-1 scrambled control peptide was used to treat NPCs for 14 days. On the other hand, short interfering RNA transfection was used to particularly knockdown the expression of Cav-1 in NPCs. Cav-1 and control siRNA were obtained from Invitrogen [53]. The Cav-1 Stealth™ RNAi provides non-overlapping Stealth™ RNAi duplex for this gene to obtain high knock-down efficiency. The duplex was transfected into NPCs with Lipofectamine RNAiMAX (Invitrogen). The Cav-1 stealth™ RNAi duplex was added at a concentration of 13 nM. A stealth™ RNAi negative control duplex (Invitrogen) was used as control. To address whether roles of Cav-1 in the inhibition of NPCs is related to VEGF dependent signaling pathway, a VEGF inhibitor (V1, 12 µM) was also used to treat NPCs prior to transfection of Cav-1 Stealth™ RNAi.

### Immunocytochemistry

The isolated cultured NPCs were plated on the coverslips and fixed in fresh 4% paraformaldehyde (in PBS, pH 7.2) at room temperature for 20 min and washed 3 times with PBS (pH 7.2). The cells were blocked and permeabilized in 10% normal goat serum plus 0.1% Triton X-100 for 1 h at room temperature. After blocking, the cells were washed with PBS and then incubated in primary antibody in PBS containing 10% normal goat serum at 4°C overnight. Primary antibodies included Cav-1 (Rabbit, 1∶400, Cell Signal), VEGF (Rabbit, 1∶50, Santa Cruz), GFAP (Rabbit, 1∶800, Millipore), Ki-67 (Rabbit, 1∶500, Abcam), Cleaved caspase-3(Asp175) (Rabbit, 1∶500, Cell Signal), Tublin β-III (Tuj-1) (Mouse, 1∶300, Covance) and NF200 (Mouse, 1∶800, Sigma). Negative control groups were simultaneously performed by omitting primary antibodies to exclude the presence of non-specific immunofluorescent staining. After rinsing with PBS, the coverslips were incubated with appropriate species-specific Alexa Fluor 488- or 568-conjugated IgG (1∶200, Invitrogen) antibodies in the dark at room temperature for 1 h. The coverslips were then incubated in nuclei counterstained with DAPI (4′-6-Diamidino-2-phenylindole, 1 µg/ml) and mounted in fluorescent mounting medium (Dako). The fluorescence imaging was visualized by using a Carl Zeiss Axio Observer Z1 fluorescent imaging system. The images obtained were analyzed by mean of Image J software (NIH, USA).

### Western blot analysis

Proteins were extracted from cell pellets and dissolved in RIPA cell lysis buffer containing a protease inhibitor cocktail and a phosphatase inhibitor cocktail (Sigma). Protein concentrations were determined with a Bradford protein assay kit (Bio-Rad). The proteins was denatured with reducing buffer (Laemmli) for 10 min and separated through 8–12% SDS-PAGE gels. After electrophoresis, the proteins were transferred to polyvinylidene difluoride (PVDF) membrane by electrophoretic transfer. The membranes were blocked in 5% BSA for 2 h, rinsed, and incubated overnight at 4°C with primary antibodies in 5% BSA. Primary antibodies included Tublin β-III (Tuj-1, Mouse, 1∶1000, Covance), nestin (Mouse,1∶1000, BD), flk1 (Mouse, 1∶800, Sigma), phospho-p44/42MAPK (Thr202/Tyr204) (Rabbit, 1∶1000, Cell Signal), phospho-Stat3 (Tyr705) (Rabbit, 1∶1000, Cell Signal), phospho-Stat3 (Ser727) (Rabbit, 1∶1000, Cell Signal), phospho-Akt (Ser473) (Rabbit, 1∶1000, Cell Signal) and Cav-1 (Rabbit, 1∶1000, Cell signal). Excess antibodies were removed by washing the membrane with TBS/0.1% Tween-20 and the membranes were incubated for 1 hr with horseradish peroxidase-conjugated secondary antibodies. To normalize the amounts of proteins applied to SDS-PAGE, the membranes initially incubated with primary and secondary antibodies, were reprobed with β-actin (Mouse, 1∶2000, Sigma), p44/42MAPK (Rabbit, 1∶1000, Cell signal), Akt (Rabbit, 1∶1000, Cell signal) and Stat3 (Rabbit, 1∶1000, Cell signal) antibodies as internal references, respectively. The bands were visualized by advanced chemoluminescence (GE, Healthcare Life Sciences), recorded by Gel-Doc (Bio-Rad) and the relative band intensity was quantified by Quantity One software (Bio-Rad).

### ELISA assay

Cell culture supernatants were collected at 0, 1, 3, 7 and 14 days (n = 3, at each time point). A VEGF immunoassay kit (R&D systems) was used according to manufacturer's protocol. Absorbance was determined using a Bio-Rad microplate reader at 450 nm, reference wavelength at 540 nm.

### Statistical analysis

All data were presented as means ± S.D. Comparisons of multiple groups were done by one-way analysis of variance (ANOVA) dependent experimental designs and followed by Student-Newman-Keuls (S-N-K) test for two group comparisons within the multiple groups with SPSS 16.0 statistical programs. In two group designed experiments, comparisons were done by using unpaired student's t-test. Significance was set as a probability level of p<0.05.

## Supporting Information

Figure S1 **Western blot analysis on the phosphorylation of Stat3 in NPCs under normoxic and hypoxia-reoxygenation conditions.** For hypoxia-reoxygenation treatment, NPCs were exposed 1% to O_2_ for 24 h and then switched to 21% O_2_ for 1, 3, 7, and 14 days, while for normoxic treatment, NPCs consistently cultured under normoxia with 21% O_2_. **A. Representative immunoblot detection for phosphorylation of Stat3.** Cell lysates were blotted with p-Stat3 (Ser 727) antibody, in which Stat3 were used as internal reference. **B. Statistical analysis on the phosphorylation of Stat3** (Mean ± S.D., n = 3). Phosphorylation of Stat3 was presented as fold activation of light units normalized to Stat3. Observed day versus day 1 under the same oxygenation condition, ## p<0.01.
(TIF)

Figure S2 **Effects of Cav-1 scaffolding domain peptide on the phosphorylation of Stat3 in NPCs under normoxic condition.** Phosphorylation of Stat3 in NPCs at day 14 were analyzed with western blot analysis. Cell lysates were blotted with p-Stat3 (Ser 727) antibody, in which Stat3 was used as internal reference. **A. Representative immunoblot detection for phosphorylation of Stat3.** Blank, blank control group; Con P, Cav-1 scrambled control peptide group; Cav P, Cav-1 peptide group. **B. Statistical analysis on the phosphorylation of Stat3** (Mean ± S.D., n = 3). The phosphorylation of Stat3 was presented as fold activation of light units normalized to Stat3. Cav P versus blank, ** p<0.01; Cav P versus Con P, ## p<0.01.
(TIF)

Figure S3 **Effects of siRNA Cav-1 specific knockdown on the phosphorylation of Stat3 in NPCs with or without V1 treatment under normoxic condition.** A short interfering RNA transfection was used to knock down the expression of Cav-1 in NPCs. NPCs were transfected with Cav-1 Stealth™ RNAi. All data were obtained at day 14. **A. Representative immunoblot detection for phosphorylation of Stat3 in NPCs treated by Cav-1 specific knockdown with or without V1 treatment** (Mean ± S.D., n = 3). VEGF inhibitor V1 (12 µM) was used to treat NPCs prior to transfection of Cav-1 Stealth™ RNAi. Cell lysates were blotted with the p-Stat3 (Ser727) antibody, in which Stat3 was used as internal reference. NC, negative control group; siCav-1, Cav-1 RNA silencing group; siCav-1+V1: Cav-1 RNA silencing+V1 group; **B. Statistical analysis on the phosphorylation of Stat3** (Mean ± S.D., n = 3). The phosphorylation of Stat3 was presented as the fold activation of light units normalized to Stat3. Cav-1 siRNA versus negative control, ** p<0.01; Cav-1 siRNA+V1 versus Cav-1 siRNA, ## p<0.01.
(TIF)
