# Supplementary figures and images for: Caveolin-1 Plays a Crucial Role in Inhibiting Neuronal Differentiation of Neural Stem/Progenitor Cells via VEGF Signaling-Dependent Pathway
Source: PLoS One. 2011 Aug 3;6(8):e22901. doi: 10.1371/journal.pone.0022901 (PMC3149620; doi:10.1371/journal.pone.0022901)

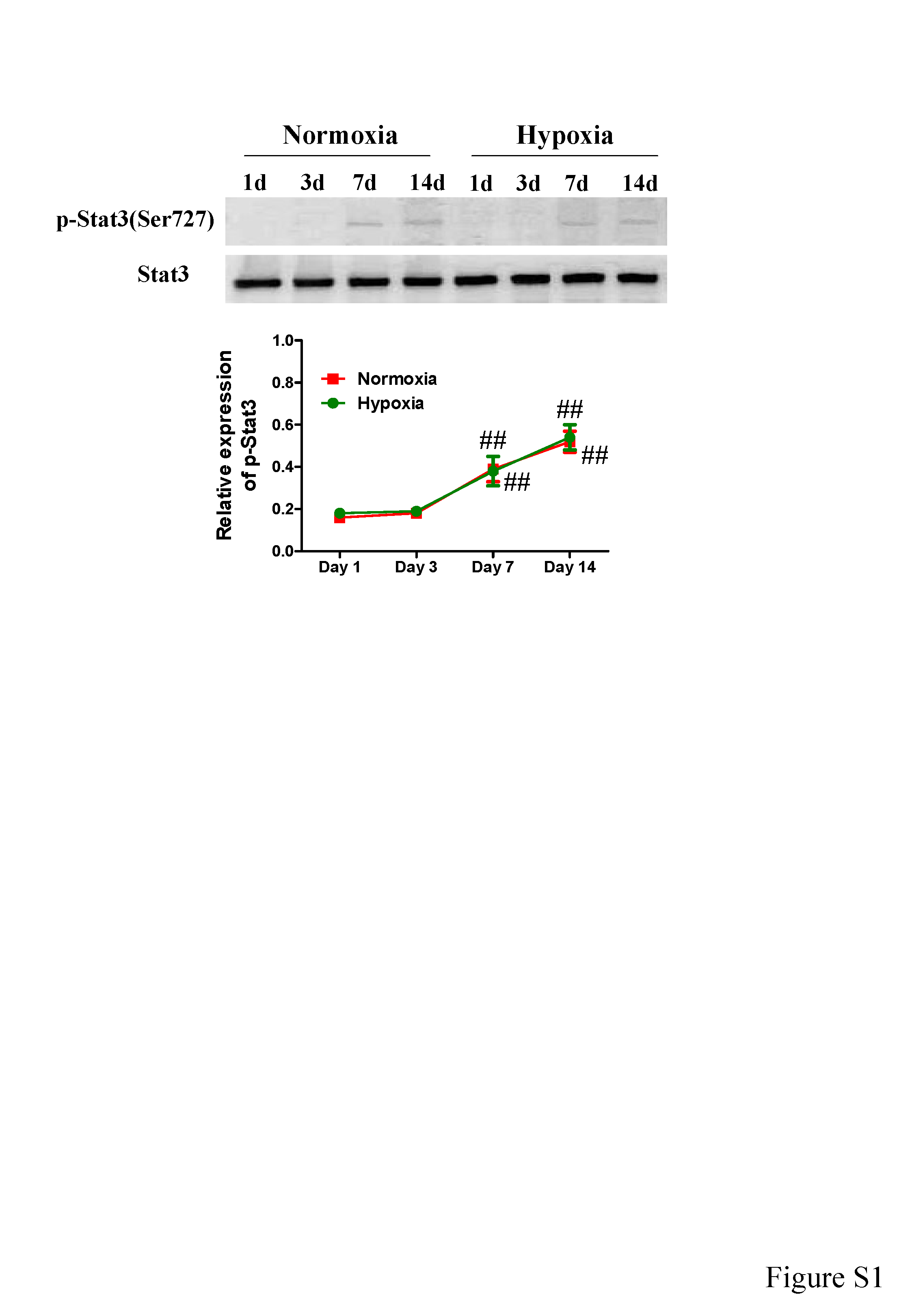

Supplement: Figure S1 — Western blot analysis on the phosphorylation of Stat3 in NPCs under normoxic and hypoxia-reoxygenation conditions. For hypoxia-reoxygenation treatment, NPCs were exposed 1% to O2 for 24 h and then switched to 21% O2 for 1, 3, 7, and 14 days, while for normoxic treatment, NPCs consistently cultured under normoxia with 21% O2. A. Representative immunoblot detection for phosphorylation of Stat3. Cell lysates were blotted with p-Stat3 (Ser 727) antibody, in which Stat3 were used as internal reference. B. Statistical analysis on the phosphorylation of Stat3 (Mean ± S.D., n = 3). Phosphorylation of Stat3 was presented as fold activation of light units normalized to Stat3. Observed day versus day 1 under the same oxygenation condition, ## p<0.01. (TIF) [file pone.0022901.s001.tif]

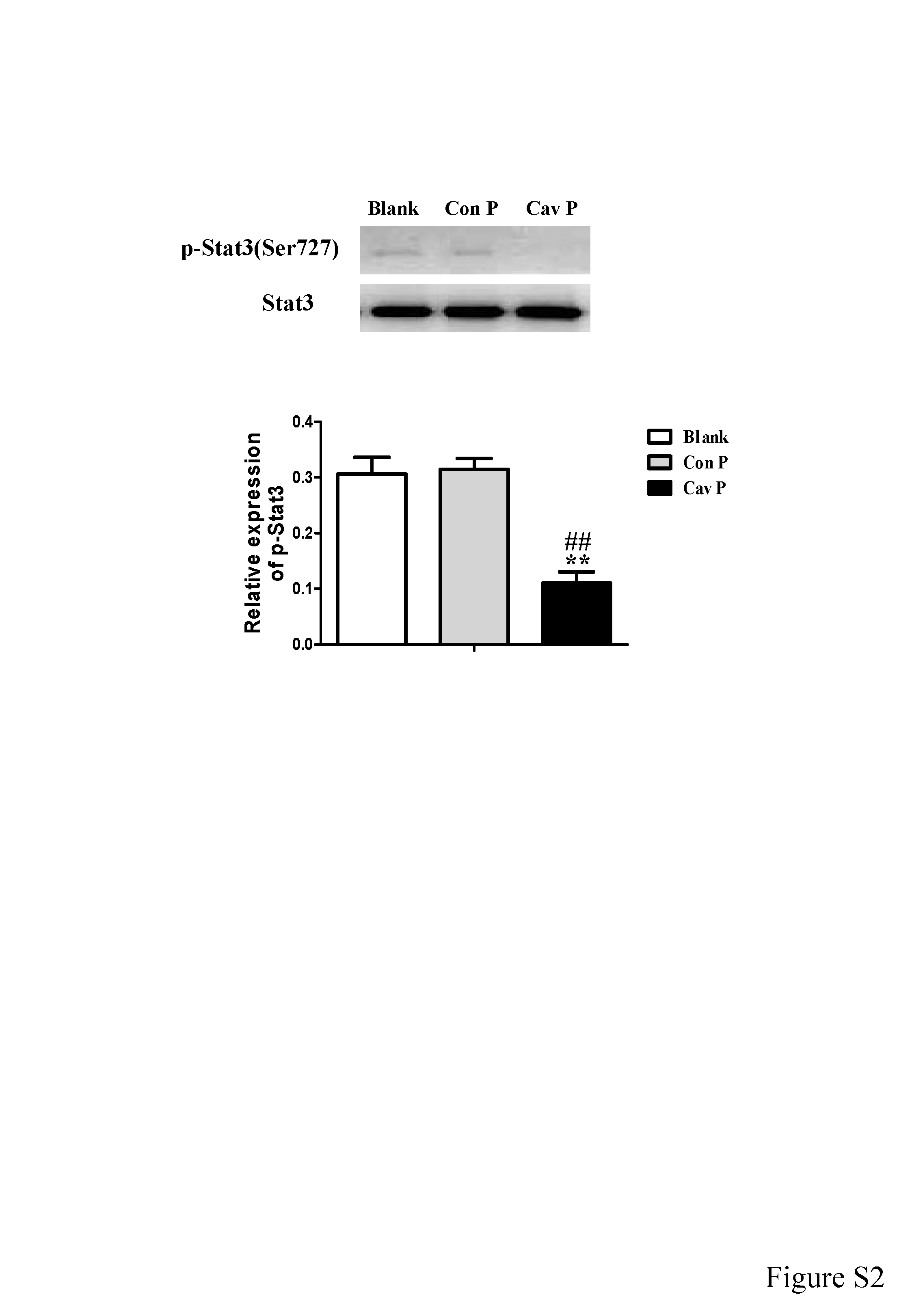

Supplement: Figure S2 — Effects of Cav-1 scaffolding domain peptide on the phosphorylation of Stat3 in NPCs under normoxic condition. Phosphorylation of Stat3 in NPCs at day 14 were analyzed with western blot analysis. Cell lysates were blotted with p-Stat3 (Ser 727) antibody, in which Stat3 was used as internal reference. A. Representative immunoblot detection for phosphorylation of Stat3. Blank, blank control group; Con P, Cav-1 scrambled control peptide group; Cav P, Cav-1 peptide group. B. Statistical analysis on the phosphorylation of Stat3 (Mean ± S.D., n = 3). The phosphorylation of Stat3 was presented as fold activation of light units normalized to Stat3. Cav P versus blank, ** p<0.01; Cav P versus Con P, ## p<0.01. (TIF) [file pone.0022901.s002.tif]

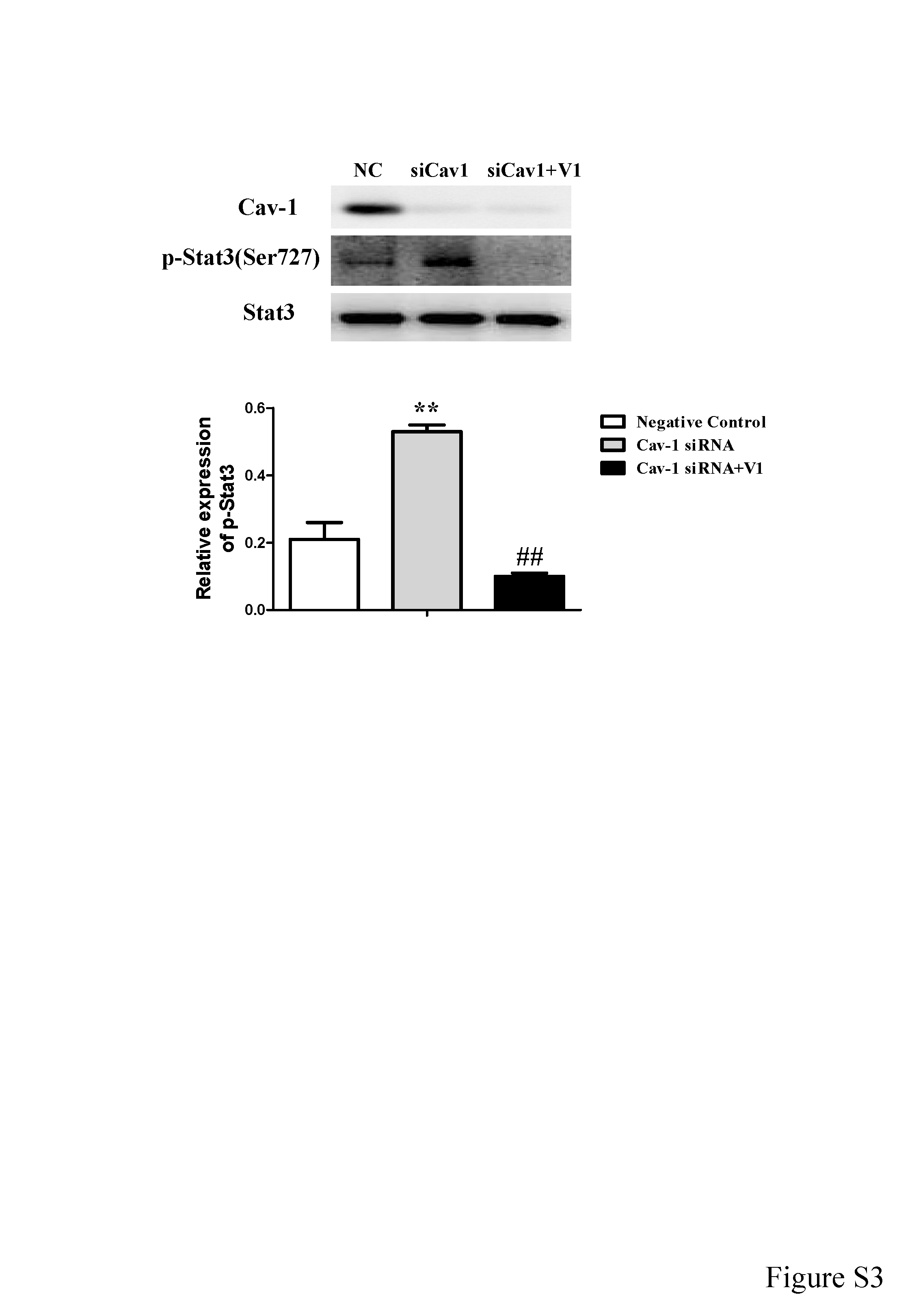

Supplement: Figure S3 — Effects of siRNA Cav-1 specific knockdown on the phosphorylation of Stat3 in NPCs with or without V1 treatment under normoxic condition. A short interfering RNA transfection was used to knock down the expression of Cav-1 in NPCs. NPCs were transfected with Cav-1 Stealth™ RNAi. All data were obtained at day 14. A. Representative immunoblot detection for phosphorylation of Stat3 in NPCs treated by Cav-1 specific knockdown with or without V1 treatment (Mean ± S.D., n = 3). VEGF inhibitor V1 (12 µM) was used to treat NPCs prior to transfection of Cav-1 Stealth™ RNAi. Cell lysates were blotted with the p-Stat3 (Ser727) antibody, in which Stat3 was used as internal reference. NC, negative control group; siCav-1, Cav-1 RNA silencing group; siCav-1+V1: Cav-1 RNA silencing+V1 group; B. Statistical analysis on the phosphorylation of Stat3 (Mean ± S.D., n = 3). The phosphorylation of Stat3 was presented as the fold activation of light units normalized to Stat3. Cav-1 siRNA versus negative control, ** p<0.01; Cav-1 siRNA+V1 versus Cav-1 siRNA, ## p<0.01. (TIF) [file pone.0022901.s003.tif]
